# Endometrial receptivity in cattle: the mutual reprogramming paradigm

**DOI:** 10.1590/1984-3143-AR2022-0097

**Published:** 2022-12-16

**Authors:** Mario Binelli, Felipe Alves Correa Carvalho Silva, Cecilia Constantino Rocha, Thiago Martins, Mariana Sponchiado, Veerle Van Hoeck, Andrey Cordeiro, Meghan Campbell, Jo L. M. R. Leroy, Francisco Peñagaricano, Guilherme Pugliesi

**Affiliations:** 1 Department of Animal Sciences, University of Florida, Gainesville, Florida, USA; 2 Department of Animal and Dairy Sciences and Brown Loam Experiment Station, Mississippi State University, Raymond, Mississippi, USA; 3 Department of Physiological Sciences, College of Veterinary Medicine, University of Florida, Gainesville, Florida, USA; 4 Kemin Europa, Animal Nutrition and Health EMENA, Toekomstlaan, Herentals, Belgium; 5 Centro de Ciências Biológicas e da Natureza, Universidade Federal do Acre, Rio Branco, AC, Brasil; 6 Department of Veterinary Sciences, Gamete Research Centre, University of Antwerp, Wilrijk, Belgium; 7 Department of Animal and Dairy Sciences, University of Wisconsin-Madison, Madison, Wisconsin, USA; 8 Departamento de Reprodução Animal, Faculdade de Medicina Veterinária e Zootecnia, Universidade de São Paulo, Pirassununga, SP, Brasil

**Keywords:** cattle, endometrium, sex-steroids, embryo

## Abstract

Prior to implantation in cattle, the mucous medium contained in the uterine lumen serves as a working interface for molecular exchange and signaling between the lining endometrium and the embryo. The composition of this luminal fluid changes temporally according to the secretory and reabsorptive activities of the uterus and the embryo, which are under complex regulation. Via this interface, both the embryo and the endometrium reprogram each other’s functions to support pregnancy continuation beyond the pre-implantation period. More specifically, the embryo receives elongation signals and the uterus receives anti-luteolytic stimuli. Here, characteristics of the luminal compartment as well as the regulation of its composition to determine the pregnancy outcome will be discussed.

## Introduction - the mutual reprogramming paradigm

**Figure 1:**
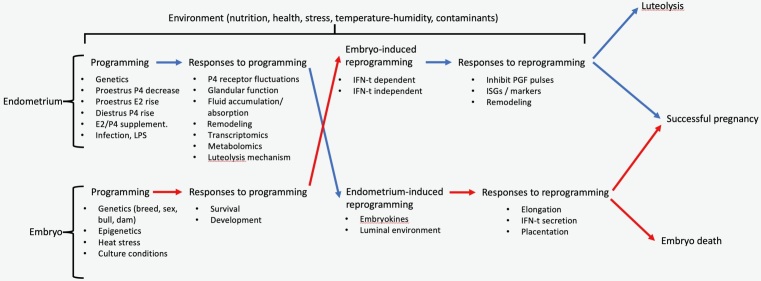
The mutual reprogramming paradigm for pre-implantation gestational success in cattle. Initial endometrial and the embryonic function are in response to both intrinsic (e.g., genetics) and extrinsic (e.g., sex-steroids [P4; progesterone, E2; estradiol], environment) stimuli. Default endometrial programming will lead to the production of luteolytic pulses of prostaglandin F2α (PGF) and the consequent regression of the corpus luteum. Embryo development outside of the reproductive tract (i.e., in vitro) stops prior to elongation. In vivo, mutual reprogramming stimuli between the embryo and the uterus are required for pre-implantation pregnancy to succeed. Molecular exchange needed for reprogramming takes place in the luminal fluid. LPS: Lipopolysaccharide, IFN-t : Interferon-tau, ISGs: Interferon stimulated genes.

In cattle, pregnancy losses are most frequent during the first three weeks post-estrus, the pre-implantation window (Reese et al., 2020). Such losses are attributed to dysfunctions in the maternal unit, embryonic unit, or their interaction. For the purpose of this paper, the maternal unit is considered at the level of the reproductive tract, with an emphasis on the uterus, and the embryonic unit is considered both the pre-hatching embryo and the elongating conceptus. A critical characteristic of the functional uterus is the biochemical composition of the luminal environment. Prior to implantation, embryo nutrition is histotrophic. Thus, molecules secreted by, or transported to the lumen will compose the mucous medium that surrounds the embryo. This luminal fluid also contains molecules secreted by the embryo, and therefore serves as an interface for paracrine signaling between both units. The path to a successful pregnancy involves spatial-temporal regulation of both maternal and embryonic function. Prior to embryo elongation, regulation of the uterine function is exerted mainly by fluctuations in the concentrations of the ovarian steroids, estradiol and progesterone, that occur prior to and during the pregnancy. Moreover, regulation of embryonic development is exerted by factors intrinsic to the embryo. However, at some point post-hatching (after 9-10 days post fertilization), effective cross-regulation between the uterus and the embryo is required for pregnancy success (Figure 1). Specifically, the default uterine program is to release pulses of prostaglandin F2α (PGF) starting approximately 17 days after estrus (Shirasuna et al., 2004). This will lead to luteal regression and the start of a new estrous cycle. Such default mechanism must be reprogrammed to prevent luteal regression and foster pregnancy continuation. An elongated conceptus that is capable to secrete interferon-tau (IFN-t) is required to reprogram the endometrium, that will then support the pregnancy rather than the estrous cyclicity (Thatcher et al., 2001). Likewise, in vitro, a hatched embryo will not elongate or survive (Hansen, 2020). Signaling from the endometrium is required for conceptus development towards implantation and placentation. Figure 1 summarizes the mutual reprogramming paradigm. In this paper, biochemical and biophysical characteristics of the luminal compartment during the pre-implantation period will be discussed, as well as the regulation of luminal function via different experimental models.

## The luminal compartment is the interface for pre-implantation exchange

**Figure 2:**
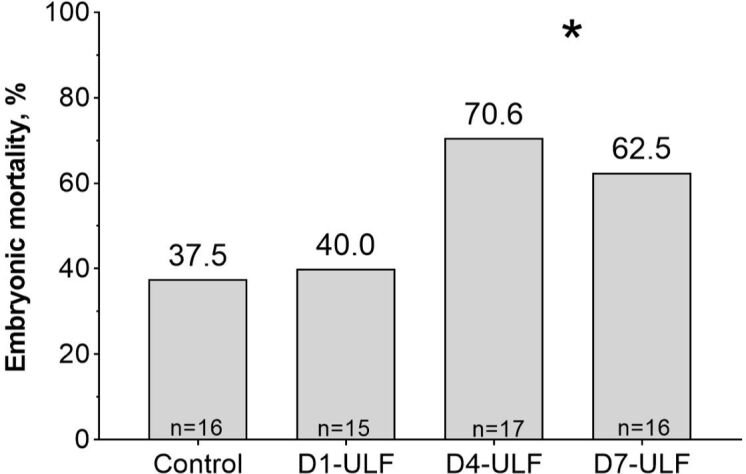
Pregnancy losses between the day of embryo transfer and 30 days after estrus in recipients submitted to uterine luminal flushings (ULF) 1, 4 or 7 days after estrus. Mortality increased sharply when washings were conducted at the later moments [Adapted from Martins et al. (2018)]. *Means significantly different (P<0.05) comparing days 4 and 7 vs. control and day 1.

In cattle, pre-implantation embryo/conceptus development occurs in the confinement of the uterine lumen. During this time, there is only a loose attachment between the embryonic and maternal tissues. Thus, most biochemical exchanges rely on the mucous luminal milieu. It follows from this concept, and from the reprogramming paradigm explained above, that the composition of the luminal environment must be essential for a successful pregnancy. Martins et al. (2018) tested this concept in a series of *in vivo* experiments. First, they synchronized cows and used a Foley catheter to gently wash the uterine lumen of both horns 1, 4, and 7 days after estrus. The objective was to remove the luminal contents, causing a global impoverishment of that cavity. Unexpectedly, when they performed a follow-up washing, they measured a nearly two-fold increase in total protein recovered from the lumen. Furthermore, Coomassie stain analysis of protein gels revealed a large band that corresponded to albumin, suggesting that the washings evoked blood protein influx to the luminal compartment. Such changes were taken as evidence that the uterine washings effectively modified the composition of the luminal compartment. In a second experiment, authors used the same design, except that each cow received three fresh, in vitro-produced embryos, 7.5 days after estrus, and pregnancy was measured by ultrasonography 30 days after estrus. The rationale of transferring three embryos was to minimize the chance that the pregnancy outcome would be determined by the quality of a single embryo. What they found was that embryo mortality almost doubled when washings were conducted 4 or 7 days after estrus compared to the group washed 1 day after estrus and the control group (not washed; Figure 2). They concluded that mechanically disturbing the composition of the uterine lumen led to a decreased pregnancy success, probably by changing the relative concentration of molecules in that compartment. An additional important finding was that, when washings were conducted one day after estrus, pregnancy outcome was not affected. This indicates that there were mechanisms to replenish the luminal environment after the perturbation induced by washing, that allowed the recovery of the ability of the uterus to sustain a pregnancy. One such mechanism potentially involved in the control of the composition of the lumen is the regulation of luminal fluid accumulation and reabsorption that occurs during the peri-estrus period (Pierson and Ginther, 1987).

**Figure 3:**
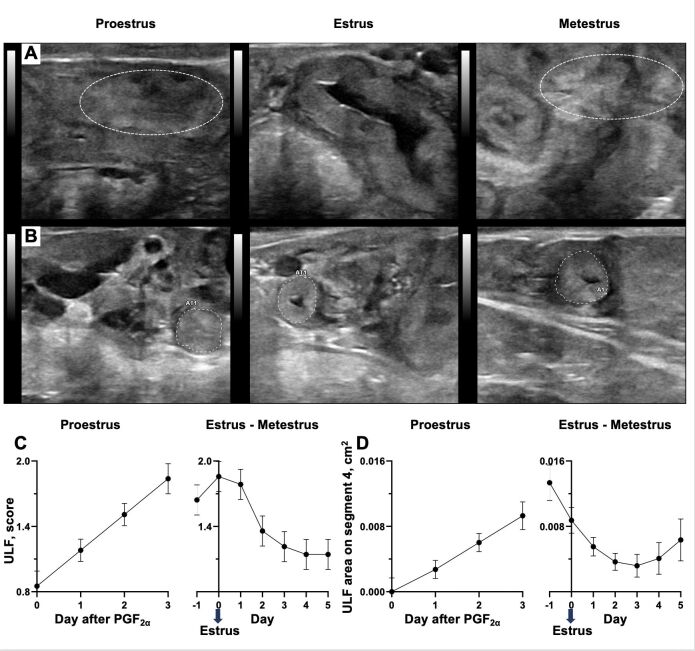
Temporal dynamics of uterine variables at proestrus, estrus, and metestrus. Sonograms used for scoring the uterine luminal fluid (ULF) accumulation on the entire organ (A) and for measuring the area of uterine luminal fluid accumulation on segment 4 of the uterine horn (B). Notice that patterns of fluid accumulation differ regionally in the uterus especially at metestrus when accumulation is minimum in the uterus as a whole but remains present at segment 4. Average temporal changes in the uterine luminal fluid score of the entire organ (C) and on segment 4 of the uterine horn [D; N=14, Adapted from Silva et al. (2021)].

Pierson and Ginther (1987) described a change in the accumulation of fluid in the uterus of heifers, which increased during proestrus and estrus and decreased after ovulation, to remain at nadir levels throughout the diestrus. Recently, Silva et al. (2021) revisited this phenomenon to confirm and expand the previous report by Pierson and Ginther. Silva and co-authors injected heifers with PGF to induce regression of the corpus luteum and monitored them daily for signs of estrus. From the day of PGF injection to 5 days after estrus, the accumulation of fluid in the uterus was measured daily by ultrasonography in two locations. A “uterine luminal fluid score” was attributed after subjective evaluation of the amount of fluid present considering the entire organ. Also, the uterine luminal area containing fluid was measured at the uterine segment 4 of the uterine horn, which is the most cranial region onto which an ultrasound image could be taken. The main findings were as follows: (1) the temporal patterns of uterine fluid accumulation and reabsorption described previously by Pierson and Ginther were confirmed; (2) there is a very clear variation in the magnitude of fluid accumulation among animals; (3) there was an unexpected change in the pattern of fluid reabsorption 5 days after estrus for the two regions assessed. Specifically, while uterine luminal fluid scores remained at minimal levels, the area containing fluid increased in segment 4 (Figure 3). From a global perspective, the massive reabsorption of luminal fluid occurring soon after estrus likely adjusts the concentration of molecules remaining in the lumen. Perhaps such adjustment allowed the lumen of cows washed one day after estrus in Martins et al. (2018) to recover its ability to support pregnancy. From a local perspective, the increased fluid accumulation in segment 4 five days after estrus coincided with the moment of embryo arrival in the uterus, had the animals been pregnant. Altogether, local and global temporal patterns of luminal fluid accumulation and reabsorption add a layer of regulation in the concentration of molecules potentially participating in the maternal-embryonic crosstalk, and thereby defining the pregnancy outcome.

Evidence to characterize the lumen as a working interface between the embryo and the uterus was presented in Belaz et al. (2016). Authors collected endometrium and PBS-luminal washings post-mortem, on days four and seven of the estrous cycle, from cows treated to ovulate a smaller follicle or a larger follicle as described by Mesquita et al. (2014). Manipulations increased proestrus concentrations of estradiol and diestrus concentrations of progesterone for the group that ovulated a larger follicle, which has also been associated with greater fertility to artificial insemination, compared to the smaller follicle group (Pugliesi et al., 2016). Both the endometrial tissue and the luminal washings were analyzed by MALDI-mass spectrometry for the relative abundance of lipids. Within each specific day of the estrous cycle (four or seven), the abundance of specific phospholipids in the endometrium was different between groups. More importantly, treatment differences in the abundance of the same lipids in the luminal washings, were consistent with those measured in the endometrium. One likely interpretation is that the origin of lipids present in the luminal fluid is the lining endometrium. The implication is that, if present, an embryo would be surrounded by the luminal fluid and in contact with such lipids, which would be available for the embryo. Overall, sex-steroid programming of the endometrial lipid composition is reflected in the luminal fluid lipid pool and potentially the embryo lipid composition. Once again, this fits the paradigm of uterus-induced reprogramming of the embryo, via the luminal fluid interface.

## The multi-component regulation of luminal function

Luminal function is the ability of the luminal fluid to provide molecular signals (such as hormones, growth factors and cytokines (Simintiras et al., 2019a), building blocks (such as amino acids and lipids, (Belaz et al., 2016; França et al., 2017) and energy sources (such as carbohydrates (França et al., 2015; Gao et al., 2009) to both the embryo and the lining endometrium. Luminal function is synonymous to the uterine fluid composition, which is ultimately determined by the secretory and reabsorptive activities of both embryonic and endometrial tissues, and the semi-autonomous capacity of the fluid itself to change its composition (Simintiras et al., 2019b). Regulation of secretory and reabsorptive activities of the embryonic and endometrial tissues, to and from the lumen, is complex and multifactorial. The aforementioned study by Silva et al. (2021) addressed such complexity. From the day of PGF-induced luteolysis to 5 days after estrus, the authors measured daily concentrations of progesterone, luteal and the largest follicular size and blood perfusion, uterine fluid accumulation, endometrial blood perfusion and thickness. The average temporal patterns of changes in these variables were as expected. However, patterns of individual heifers varied widely. Then, the authors took a multivariate approach and interrogated all variables mentioned above on a principal component analysis. The main result was that heifers were grouped in two clusters, which were associated with the first principal component. In general, clustering was based on the temporal dynamics of change in the endometrial thickness, fluid accumulation and progesterone concentrations. In that same study, researchers used a cytology brush (Cardoso et al., 2017) to collect sample from the luminal fluid at the uterine body of each animal, four days after estrus. Luminal contents were analyzed by liquid chromatography and mass spectrometry for targeted metabolite concentrations. The authors analyzed metabolite concentrations using a multivariate approach and determined that the clustering of animals generated by the metabolomic analysis was almost identical to the clustering generated by the hormonal, uterine and ovarian analyses. In summary, animals that presented a greater accumulation of uterine fluid, smaller change in endometrial thickness and decreased concentrations of progesterone had a greater concentration of amino acids in the luminal fluid compared to animals with a contrasting hormonal, uterine and ovarian phenotype. Interpretation of the functional meaning of such contrasting concentrations of amino acids in the uterine lumen is unclear. On one hand the available nutrients may be beneficial to the embryo, which would be arriving at the uterus a day later, had there been a pregnancy. On the other hand, less amino acids in the lumen may mean that these molecules were used by the uterus, in processes such as cell proliferation, which are necessary to better support embryo needs. The main point here is that changes in the composition of the luminal fluid that may affect the pregnancy are associated with complex phenotypes, that involve temporal changes in multiple variables, which are subjected to multiple points of control.

**Figure 4:**
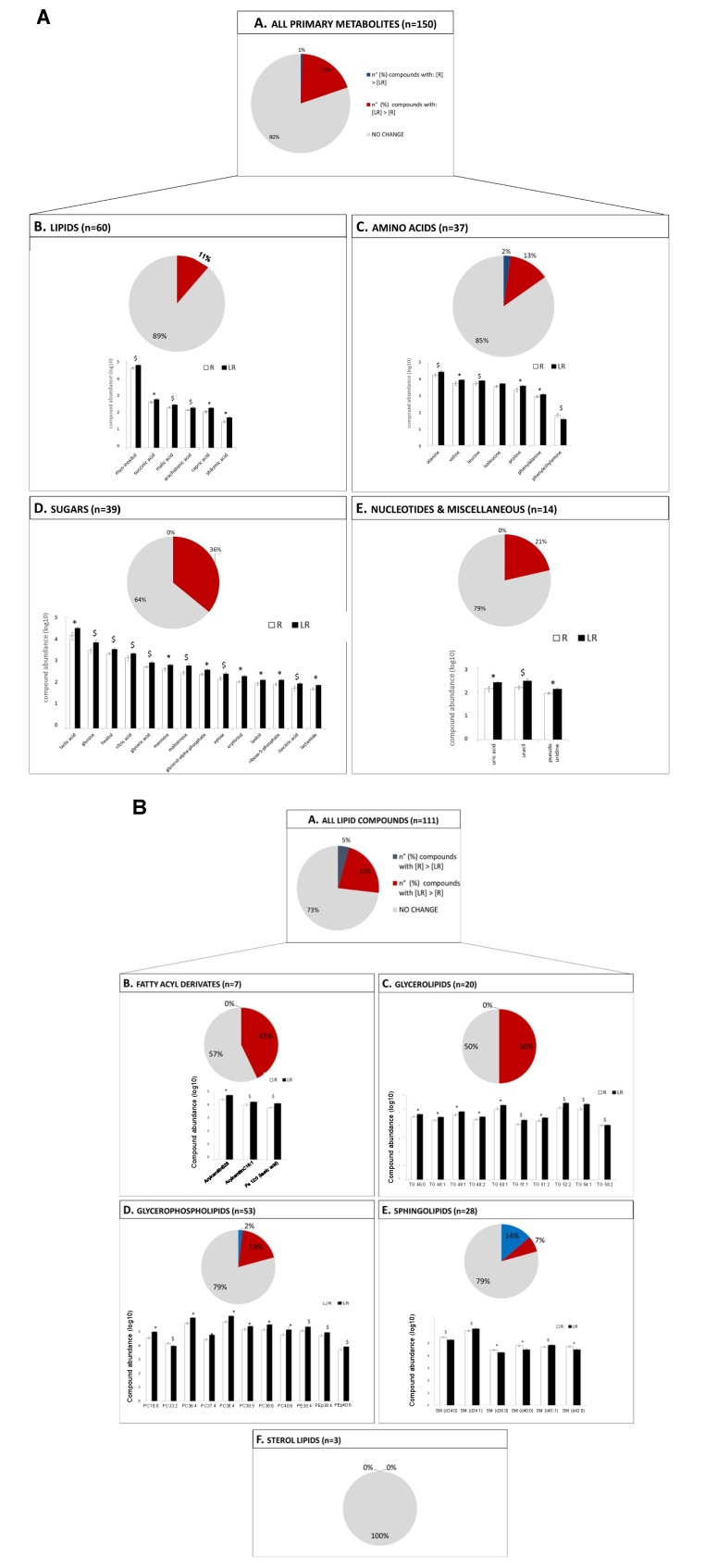
Abundance of primary metabolite (A) and lipids (B) in luminal flushings collected post-mortem, at day four of the estrous cycle, from cows treated to ovulate a larger follicle (“receptive” luminal environment; R) or a smaller follicle (“low-receptivity” luminal environment; LR). For both A and B: Panel A. Total number of primary metabolites/lipids that displayed receptivity-related abundance (P<0.1) in the D4 uterine flushing. Panel B, C, D & E. Assignment of receptivity-associated compounds (P<0.1) to main biochemical metabolite and lipid classes. For pie-charts: (grey; ■) % for compounds with no receptivity-linked differences in concentrations; (blue; ■) % of compounds with greater abundance in R uterine flushing vs. LR uterine flushing; (red; ■) % of compounds with greater abundance in LR uterine flushing vs. R uterine flushing. For bar-charts: (R; □) receptive cows and (LR; ■) low receptive cows; bar represents mean ± standard error of the mean; Metabolite and lipid data were log10 transformed before plotting. Group means of compounds with * superscript are significantly different (P<0.05); Group means of compounds with $ superscript approached significance (P<0.1).

Fluctuations in the concentrations of estradiol and progesterone around estrus affect uterine function and fertility. This proposition has been examined in experiments such as those that supplemented estradiol in the proestrus (Sá et al., 2017), compared animals with contrasting estradiol concentrations at estrus (Jinks et al., 2013), manipulated progesterone concentrations prior to luteolysis of the previous cycle (Silva et al., 2022 unpublished data), supplemented progesterone in the diestrus (Pugliesi et al., 2016; Simintiras et al., 2019a) and manipulated pre-ovulatory follicle growth to affect both proestrus estradiol and diestrus progesterone concentrations (Mesquita et al., 2014), among others. We have used the larger vs. smaller pre-ovulatory follicle size model (Mesquita et al., 2014) to ascertain the effects of peri-estrus sex-steroid changes on endometrial functions associated with embryo development, such as proliferation and apoptosis (Mesquita et al., 2015), extracellular matrix remodeling (Scolari et al., 2017), redox environment (Ramos et al., 2015) and amino acid availability (França et al., 2017). An unexpected finding of this model was the overall reduction in the luminal concentration of metabolites of several biochemical classes at early diestrus for animals that were manipulated to ovulate a larger follicle (i.e., greater concentrations of estradiol in the proestrus and progesterone in the diestrus and greater fertility to artificial insemination; [Mesquita et al., 2014; Pugliesi et al., 2016 (Figure 4)]. Uterine luminal washings were collected post-mortem, on day four of the estrous cycle, from cows treated to ovulate larger or smaller follicles. Washings were analyzed using ultra-performance liquid chromatography (UPLC) followed by mass spectrometry (MS) for the relative abundance of primary metabolites and a combination of UPLS-MS and gas chromatography-MS for lipids. On the primary metabolite analysis, the abundance of 11, 13, 36, and 21% of lipids, amino acids, sugars and nucleotides and miscellaneous metabolites was greater in the luminal washings from cows that ovulated smaller follicles, a phenotype associated with lower receptivity to the embryo, respectively. Similarly, in the lipidomic analysis, the abundance of 43, 50, 19 and 7% of fatty acyl derivatives, glycerolipids, glycerophospholipids and sphingolipids was also greater for the same group, respectively. We interpreted these findings to mean that metabolites present in lesser concentrations in the lumen had been utilized by the intensely proliferating endometrium in the larger follicle group, as documented by Mesquita et al. (2015). We speculate that the potential consequences of this luminal phenotype include adequate support of subsequent endometrial function to support embryo development and adjustment of the quantity of select nutrients available to the embryo. Indeed, there is recent evidence to support the notion that excess nutrient availability is detrimental to embryo development (Santos et al., 2021). Finally, we recently conducted repeated *in vivo* sampling of the uterine lumen of cows, 4, 7, and 14 days after estrus, and measured the luminal concentration of select metabolites. In general, we determined that there was a sharp accumulation of metabolites, specially, phospholipids, in the lumen from seven to fourteen days after estrus. Had these cows been pregnant, increased luminal availability of lipids could serve to support cell membrane synthesis of the rapidly proliferating trophectoderm of the elongating conceptus.

Altogether, the following sequence of regulatory steps is proposed: (1) proestrus estradiol from preovulatory follicle signals to receptors in the endometrium to stimulate cell proliferation; (2) proliferating cells utilize energy sources and building blocks such as lipids, phospholipids and amino acids for endometrial cell membrane and protein synthesis and this prevents accumulation of metabolites in the lumen; (3) renewed and remodeled endometrium responds to differentiation stimulus provided by increasing progesterone signaling during diestrus; (4) the differentiated endometrium has increased secretory activity towards the lumen to promote conceptus elongation; (5) the growing conceptus secretes increasing amounts of interferon-tau to the lumen; (6) interferon-tau reprograms endometrial function to block luteolysis (the default program during the estrous cycle) and support pregnancy maintenance.

As a final point regarding the regulation of endometrial function: a lot less is known about sex-steroid-independent mechanisms of control. Martins and co-authors used the cytobrush to collect endometrial luminal epithelial cells (Rocha et al., 2022) four days after a synchronized estrus in crossbred beef embryo recipient cows (Martins et al., 2022). The authors compared the luminal epithelial transcriptome of cows that maintained vs. lost pregnancy after embryo transfer. Moreover, they mathematically adjusted gene expression to remove the effect of progesterone concentrations measured on the same day of sampling. Thus, they selected an array of genes differentially expressed between animals that succeed vs. failed to maintain pregnancy, when progesterone concentrations were held constant. They reported that cows that remained pregnant downregulated many genes associated with immune functions such as phagocytic activity and migration of leucocytes, for example. This suggests that attenuated immune activity is a characteristic of the receptive endometrium that is regulated independently of progesterone. Also, because all cows in the study were synchronized and showed estrus, the profile of pre-estrus progesterone and proestrus-estrus estradiol concentrations were likely similar among them. At this point, we remain ignorant of non-sex-steroid programming of luminal function, that impacts the pregnancy outcome.

## Conclusions

Here, we conceptualized the paradigm of mutual uterine-embryonic function reprogramming that occurs via the luminal mucous interface. Nutrient and signaling exchanges between the two tissues are dynamic and temporally regulated. Concerted endocrine, tissue, cellular and molecular effects determine the pregnancy outcome. Both interventional and observational experimental approaches, as well as creative techniques to sample the uterine lumen, allowed the critical interrogation of the molecular interactions taking place in the luminal compartment, as well as the mechanisms regulating the pregnancy success. Future studies should continue to survey the luminal compartment to determine structural and signaling molecular changes required for conceptus development beyond the pre-implantation period. Also, there is a need to identify factors regulating luminal function beyond sex-steroids, as they will present new opportunities for intervention.
